# p53 regulates expression of nuclear envelope components in cancer cells

**DOI:** 10.1186/s13062-022-00349-3

**Published:** 2022-12-02

**Authors:** Emanuele Panatta, Alessio Butera, Ivana Celardo, Marcel Leist, Gerry Melino, Ivano Amelio

**Affiliations:** 1 Department of Experimental Medicine, TOR, University of Rome Tor Vergata, 00133 Rome, Italy; 2 Division of Systems Toxicology, Department of Biology, University of Konstanz, Konstanz, Germany; 3 Division of in-Vitro Toxicology and Biomedicine, Department of Biology, University of Konstanz, Konstanz, Germany

## Abstract

Nuclear organisation and architecture are essential for the maintenance of genomic integrity as well as for the epigenetic regulations and gene expression. Disruption of lamin B1, major structural and functional member of the nuclear lamina, is observed in human laminopathies and in sporadic cancers, and leads to chromosomal rearrangements and alterations of gene expression. The tumour suppressor p53 has been shown to direct specific transcriptional programmes by regulating lamin A/C, however its relationship with lamin B1 has remained elusive. Here, we show that loss of p53 correlates with increased expression of members belonging to the nuclear pore complex and nuclear lamina and directly regulates transcription of lamin B1. We show that the genomic loci of a fraction of p53-dependent genes physically interact with lamin B1 and Nup210. This observation provides a possible mechanistic explanation for the p53-depedent changes of chromatin accessibility, with the consequent influence of expression and rearrangement of these genomic sites in pancreatic cancer. Overall, these data suggest a potential functional and biochemical regulatory network connecting p53 and nuclear architecture.

**Supplementary Information:**

The online version contains supplementary material available at 10.1186/s13062-022-00349-3.

## Introduction

Nuclear lamins comprise a class of intermediate filaments belonging to three families: Lamin A, B and C [12]. This particular type of intermediate filaments is the major structural and functional component on the inner nuclear membrane. Evidence has emerged for the participation of nuclear lamins to a plethora of biological processes ranging from regulation of gene transcription to more complex 3D chromatin configurations to epigenetic modifications. Disruptions or mutations in lamin coding genes are associated to severe human disorders collectively called laminopathies [5]. Aberrant expression of nuclear lamins is also observed in many cancers and are postulated to contribute to cancer evolution [9, 21, 46]. Lamins regulate DNA repair processes and alterations of protein level can result in the formation of spontaneous double strand DNA breaks and impairment to repair [14, 15]. Moreover, lamin B class has been directly linked to genomic integrity and chromosomal stability by directly regulating constitutive heterochromatinic regions. Indeed, upregulation of lamin B1 was shown to disrupt shelterin complex and thus triggering telomere instability resulting in both telomere fusion and loss in human cells [33].

The tumour suppressor TP53 is frequently mutated in the majority of human cancers [19, 35, 38]. Loss or inactivation of p53 associates with loss of genomic integrity and multiple mechanisms contributing to genomic instability are emerging [3, 23, 29]. Advanced stages of pancreatic ductal carcinoma (PDAC), characterized by the loss of p53, display increased expression of lamin B1 and positively correlates with more aggressive phenotypes [21]. P53 was also shown to induce a senescence programme by leading to the upregulation of p16 via direct interaction with lamin A/C [50], highlighting the role of nuclear lamins in the control of gene expression and cell phenotype. However, how p53 affects the expression of genes involved in the nuclear metabolism is still elusive.

Here, we suggest a potential p53-mediated gene network that could participate to regulation of p53 gene expression programme and genomic integrity. We show that p53 loss upregulates a broad number of genes belonging to the nuclear pore and nuclear lamina, including *Lamin B1* (*Lmnb1*) and *Nuclear Pore Complex 210 (Nup210)*. We show that genomic loci of a fraction of p53-dependent genes physically interact with lamin B1- and Nup210 and frequently undergo copy number alterations in pancreatic ductal carcinoma. Finally, correlative evidence suggests a clinical value for the p53-nuclear pore and p53-nuclear lamina axes in human cancers.

## Results and discussion

**Fig. 1:**
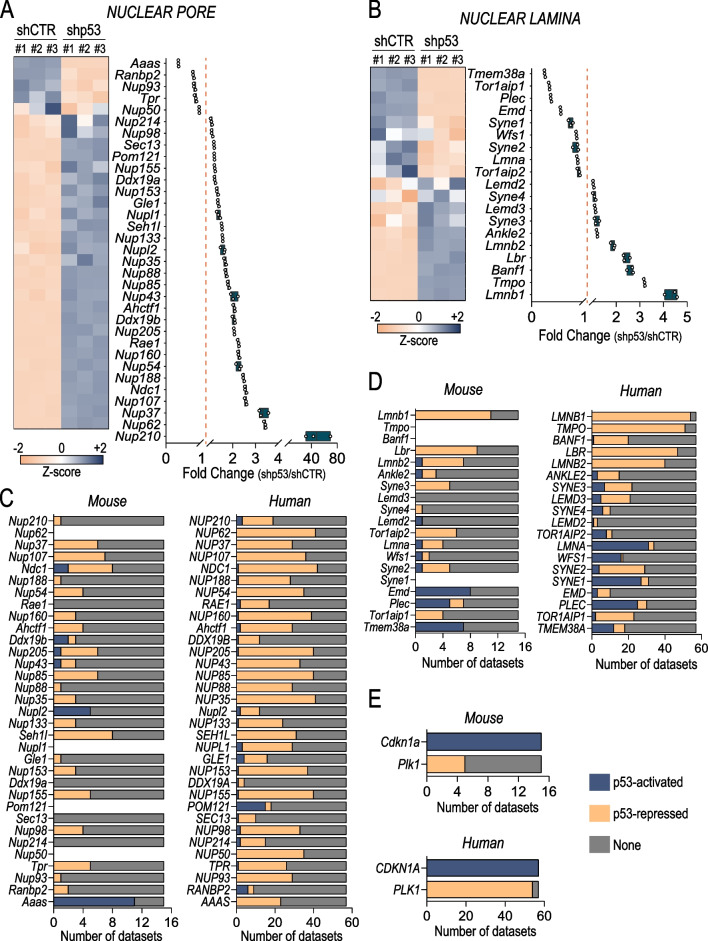
p53 transcriptionally controls the expression of nuclear envelope components. **A** and **B** RNA sequencing data of nuclear pore (**A**) and nuclear lamina (**B**). The heatmap (left) and the box plot (right) show z-score and fold change (KP shp53/KP CTR) values, respectively; the orange dotted line indicates the threshold for genes upregulated or downregulated. #1–3 are biological replicates. **C–E**, comprehensive view of p53 direct regulation on human and mouse nuclear envelope genes. CDKN1A and PLK1 are positive controls for activation and repression by p53, respectively. Source: TargetGeneRegulation database [16]

We recently showed that in PDAC-derived mouse cells p53 controls metabolic pathways by transcriptionally regulating a gene expression programme impinging on aminoacid metabolism [30]. Within this, regulation of methionine uptake appeared to affect the biosynthesis of S-adenosyl-methionine, a major methyl donor, altering the ability of the cell to cope with perturbation of DNA methylation and triggering genomic instability [6, 17, 30]. Thus, a link between epigenetic regulation of chromatin and metabolism emerged as underlying control mechanism of p53-mediated genomic integrity [27, 49]. Exploring our transcriptomic analysis of p53-depleted PDAC-derived mouse cells (KPshp53) (GSE207880), we noticed classes of nuclear proteins that displayed p53-dependent regulation, suggesting multiple levels of regulation between p53 and chromatin organisation. Specifically, we observed a negative correlation between p53 expression and genes belonging to nuclear pore, nuclear lamina, paraspekles, nucleolus and SMC complex (Fig. 1A, B and Additional file 1: Fig. S1A–C). Notably, silencing of p53 by doxycycline-inducible shRNA resulted in the upregulation of a broad number of genes belonging to the components of the nuclear compartment. To corroborate the existence of an inverse correlation between the transcription factor p53 and these genes, we interrogated the TargetGeneRegulation database. This database provides a comprehensive information of the p53-dependent gene regulatory network [16, 28]. Interestingly, a high number of mouse datasets reported the repression mediated by p53 on the nuclear lamina and nuclear pore genes. Strikingly, this repression seemed to be conserved also in humans (Fig. 1C–E and Additional file 1: Fig. S1D), supporting the relevance of this regulatory mechanism in human cancers.

**Fig. 2:**
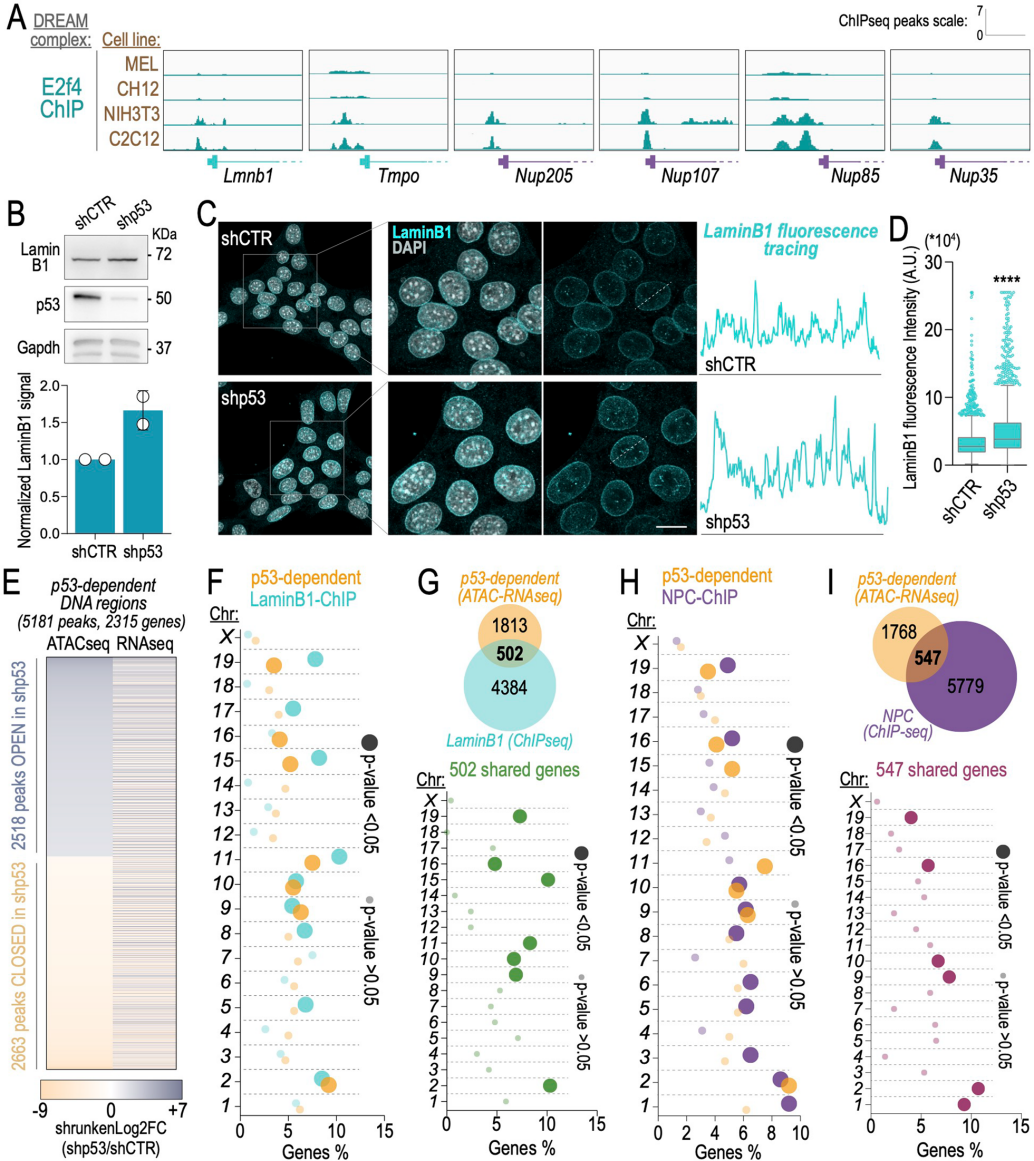
A fraction of p53-dependent genes binds to components of Nuclear Lamina and Nuclear Pore Complex. **A** ChIP-seq tracks of E2f4 in different cell lines (MEL, CH12, NIH3T3, C2C12); *Lmnb1* and *Tmpo* belong to the Nuclear Lamina; *Nup205*, *Nup107*, *Nup85*, *Nup35*, belong to the Nuclear Pore Complex. **B** immunoblot and densitometry analysis showing Lamin B1 and p53 expression in KP CTR and KP shp53 cells; a representative micrograph of 2 independent experiments is shown. GAPDH is used as loading control. **C** immunofluorescence staining (left) and fluorescent tracing (right) of Lamin B1 in KP CTR and KP shp53 cells; nuclei are stained with DAPI, scale bar, 10 µm. **D** Lamin B1 fluorescence intensity quantification; A.U. is Arbitrary Units; data are presented as boxplot with Tukey’s whiskers; ****p-value < 0.0001. **E** heatmap of ATAC and RNAseq data of 5181 p53-dependent DNA regions; data are presented as shrunken log2 fold change (FC, shp53/shCTR). **F–I** chromosome enrichment analysis of p53-dependent (**F** and **H**), Lamin B1-bound (**F**) and NPC-bound genes (**H**). 502 genes are shared between p53 and Lamin B1 (**G**), and 547 are shared between p53 and NPC (**I**); data are presented as genes percentage (%) per chromosome; *p*-value is indicated by circles diameter and colour

To gain insight into the molecular relationship between p53 and the nuclear envelope, we firstly asked whether p53 is directly involved in the regulation of the genes of interest. p53 mediates indirect repression of target genes by involving the DREAM complex [13, 39]. Thus, we analysed Chromatin Immunoprecipitation followed by sequencing (ChIP-seq) across different cell lines and we found an enrichment of DREAM factor E2f4 binding at the promoter region of *Lmnb1*, *Tmpo*, *Nup205*, *Nup107*, *Nup85* and *Nup35* (Fig. 2A).

Given the emerging role of lamins in genomic integrity [37, 40], we thus focused on lmnb1 due to its direct involvement in the chromosomal organization and heterochromatin telomeric stability. Immunoblot analysis and confocal microscopy analysis of lmnb1 showed a moderate but significant increase of lamin b1 expression upon knockdown of p53 in our KPshRNA model (Fig. 2B–D). Hence, p53 appears to directly control transcription and expression of lamin B1.

Nuclear lamins participate to the tridimensional organisation of hetero- and euchromatin by directly interacting with chromatin domains [12, 45]. Recently, the lamin B1-binding chromatin profile (lamin B1 domains) was dissected by Chromatin Immunoprecipitation followed by deep sequencing (ChIP-seq) [31]. Hence, we next performed integration of our Assay for Transposase-Accessible Chromatin followed by deep sequencing (ATAC-seq) and RNA sequencing (RNA-seq) of PDAC cells proficient/deficient for p53 (GSE207880) and identified genomic areas, whose chromatin accessibility and transcription appeared to consistently change in a p53-dependent manner (Fig. 2E). This indicated that p53-dependent gene expression appears at least in part to correlate with a p53-dependent change in chromatin organisation. Hence, we hypothesise that a correlation exists between p53-dependent chromatin regions and lamin B1-bound areas. By comparing p53-dependent regions and lamin B1-bound chromatin domains, we found 502 common genes (Fig. 2F, G), indicating that at least in part a correlation exists.

Our initial analysis indicated that also nuclear pore complex components are regulated in a p53-dependent manner (Fig. 1). The nuclear pore complex controls the nuclear metabolic reactions by regulating the shuttling of proteins between the nucleus and the cytoplasm [4, 10]. Among the nuclear pore proteins, we found Nup210 extensively upregulated following depletion of p53 (Fig. 1A). Nup210 was very recently described as a heterochromatin barrier insulator by controlling the deposition of trimethylation of lysine 27 of histone H3 (H3K27me3) [2]. Conducting Nup210 binding profile with p53-dependent chromatin reagions we found that 547 genes common genes (Fig. 2H, I). All together, these correlative analyses are suggestive of possible complementary regulatory mechanisms driven by p53 and nuclear lamina/nuclear pore complex in the regulation of p53-dependent chromatin conformation and gene expression.

**Fig. 3:**
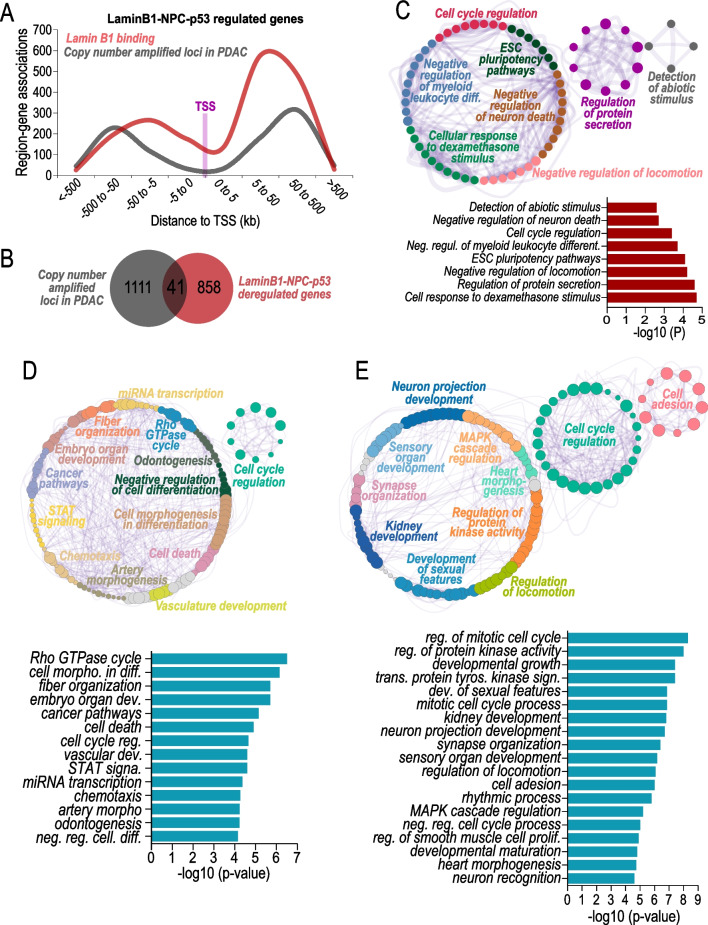
Analysis of p53-LaminB1-NPC-dependent genes. **A** LaminB1 binding (red line) and copy number alteration (grey line) relative to the transcription start site (TSS) position for the genes regulated by LaminB1-NPC-p53. **B** Venn diagram showing integration of copy number amplified loci in pancreatic ductal carcinoma (PDAC) and Lamin B1-NPC-p53-bound genes. **C** Gene ontology term analysis of the 41 genes overlapping in **B**. **C** Gene ontology term analysis of the 502 shared gene. Related to Fig. 2G. **D** and **E** gene ontology term analysis of the 547 shared genes. Cytoscape [32]

**Fig. 4:**
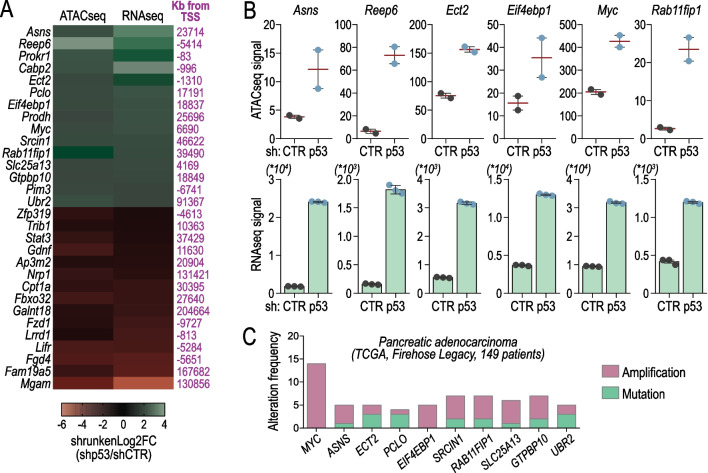
p53-Lamin B1-NPC dependent genes alterations in cancer. **A** Heatmap of ATAC and RNAseq data showing the most significant genes derived from figure 3B; data are presented as shrunken log2 fold change (FC, shp53/shCTR). **B** ATACseq signal (**Upper panel**) and RNAseq signal (**lower panel**) variation between KP CTR and KP shp53 for the indicated genes; the signal is expressed in sequencing reads; data are presented as mean (red line in **upper panel**; bar in **lower panel**) ± SEM. Dots represents biological replicates (n = 2 or n = 3); *p-value < 0.05*;* **p-value < 0.01; ****p-value < 0.0001. **C** Histogram bars showing the most recurrent mutated genes from **A** in a pancreatic cancer dataset,source: cBioPortal [8]; the genetic alteration (amplification, deletion, or mutation) frequency is expressed as percentage with respect to the cohort (308 patients)

Nuclear lamins participate to chromosomal organisation by interacting with large genomic heterochromatinic regions named lamin-associated domains (LAD) [20]. However, only recently lamins were also shown to participate to dynamic control of euchromatinic regions and regulate gene transcription [31] with potential implications for susceptibility of these loci in genomic rearrangements during cancer progression. We observed that the genomic distribution of lamin B1-NPC-p53 deregulated genes were particularly enriched between 5 and 50 Kbp from the transcription start site and some of them resulted as common genomic regions affected by copy number amplification during cancer progression (Fig. 3A, B and Additional file 1: Fig. S2). These amplified genes, including c-Myc, are involved in biological pathways such as regulation of cell cycle, pluripotency, cellular response to exogenous stimuli (Fig. 3C–E). Notably, chromatin accessibility and RNA levels of these genes were significantly increased in PDAC cells depleted of p53 and resulted commonly amplified or mutated in human pancreatic adenocarcinoma (Fig. 4A–C).

**Fig. 5:**
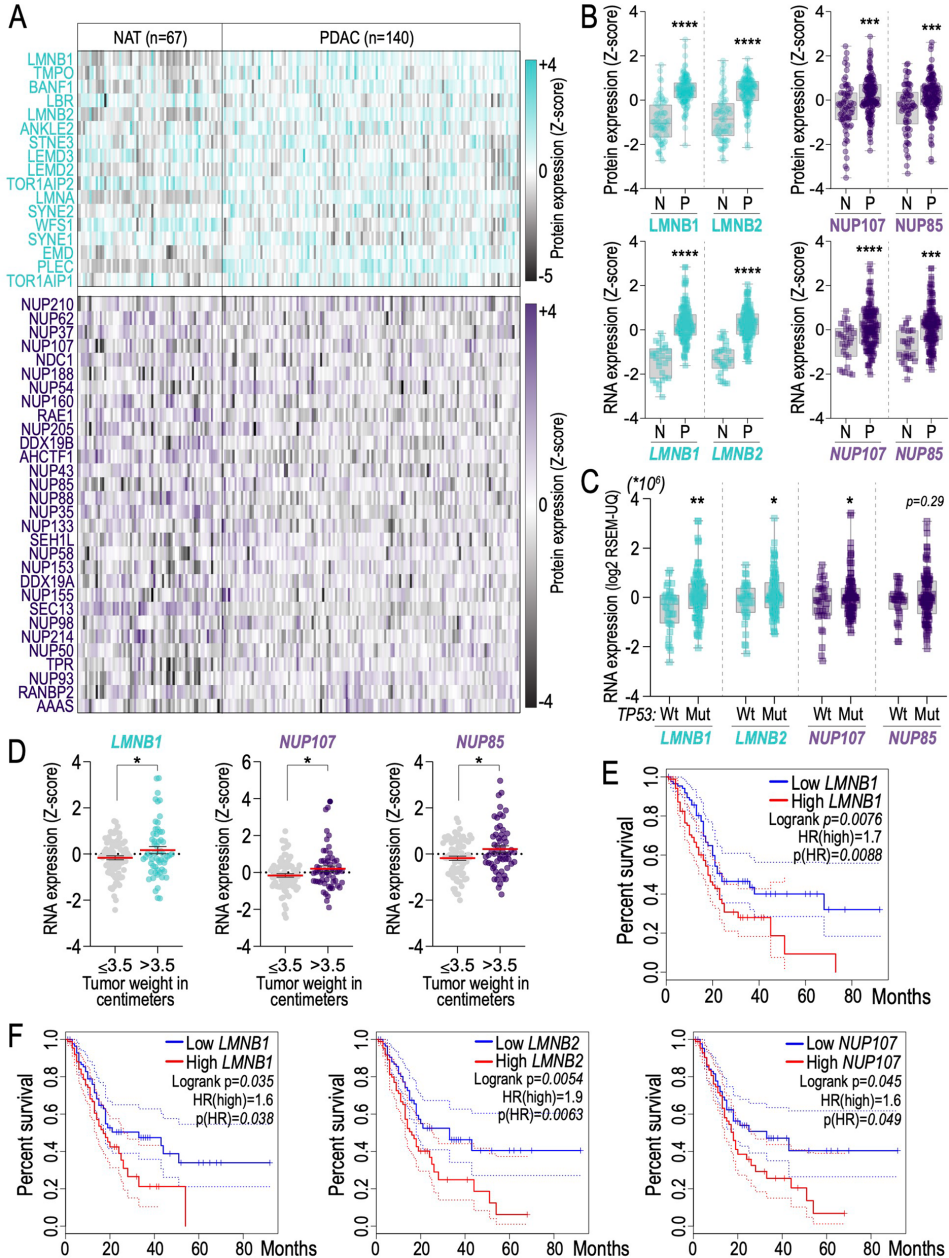
Expression of Nuclear Lamina (NL) and Nuclear Pore Complex (NPC) components is altered in PDAC and predicts prognosis. **A** Heatmap showing proteomic data of normal adjacent tissue (NAT, n = 67 patients) and pancreatic ductal adenocarcinoma (PDAC, n = 140 patients) for members of NL (top) and NPC (bottom); data are expressed as z-score. **B** protein (top) and RNA (bottom) levels between NAT (N, n = 67 patients) and PDAC (P, n = 140 patients) for selected members of NL and NPC; data are expressed as z-score by box and whiskers plot. ***p-value < 0.001; ****p-value < 0.0001. **C** RNA expression for selected members of NL and NPC according to TP53 status (wild-type, Wt; mutant, Mut); data are expressed as log2 RSEM-UQ by box and whiskers plot. *p-value < 0.05; **p-value < 0.01. **D** RNA expression of the selected members of NL and NPC in PDAC samples stratified by tumour weight (≤ 3.5 cm n = 80 patients, or > 3.5 cm n = 58 patients); dot plot data (z-score) are presented by mean (red line) ± SEM. *p-value < 0.05*.* From **A** to **D** source data is cBioPortal [8] and Cao et al. [7]. **E** overall survival of pancreatic cancer patients stratified for *LMNB1* mRNA levels. **F** disease free survival of pancreatic cancer patients stratified for *LMNB1, LMNB2, and NUP107* mRNA levels; in **E** and **F** source data is GEPIA2 (high level cohort = 89 patients; low level cohort = 89 patients)

**Fig. 6:**
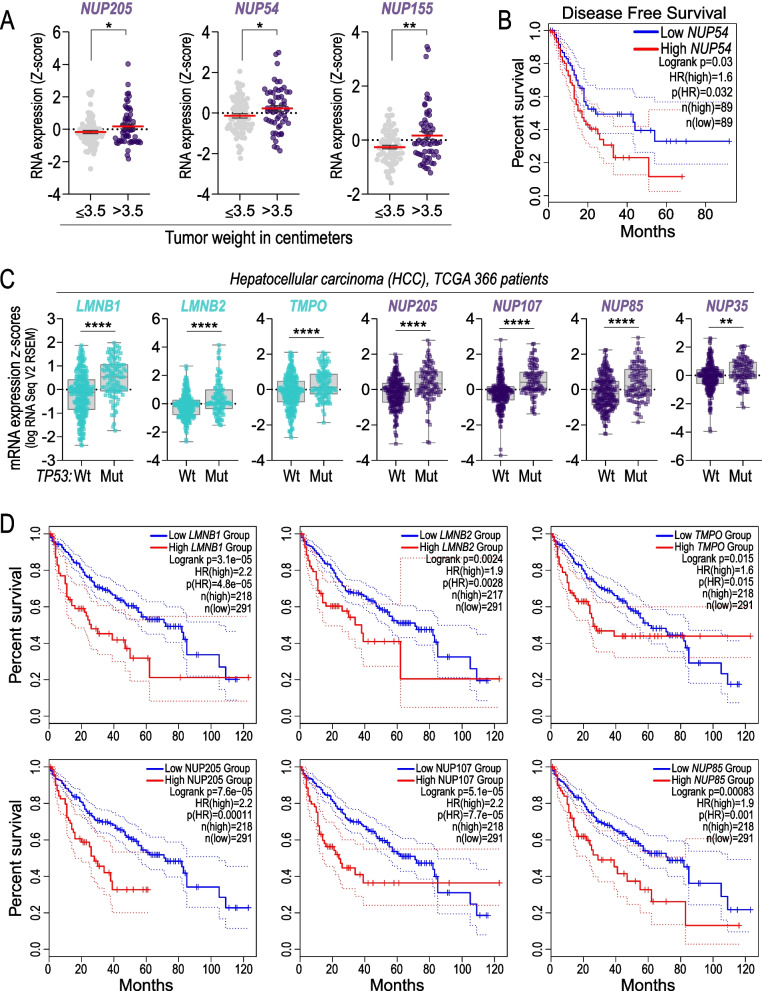
Nuclear component expression is a prognostic factor in human cancer. **A** RNA expression of the selected members of NPC in PDAC samples stratified by tumour weight (≤ 3.5 cm n = 80 patients, or > 3.5 cm n = 58 patients); dot plot data (z-score) are presented by mean (red line) ± SEM; source data: cBioportal [8], and Cao et al. [7], *p-value < 0.05; **p-value < 0.01*.* **B** Disease free survival analysis of pancreatic cancer patients stratified by *NUP54* mRNA levels; source GEPIA2 [43], (high level cohort = 89 patients; low level cohort = 89 patients). **C** RNA expression analysis for the selected members of NL and NPC according to *TP53 status,* wild type (Wt) and mutant (Mut), in hepatocellular carcinoma (HCC); data are expressed as log RNAseq V2 RSEM by box and whiskers plot; source TCGA, cBioportal [8]; n = 366 patients; **p-value < 0.01; ****p-value < 0.0001*.* **D** Overall survival of hepatocellular carcinoma (HCC) patients stratified for high or low mRNA levels of selected genes; source GEPIA2 [43]

Pancreatic ductal carcinoma shows > 70% inactivation of the tumour suppressor p53 [47, 48]. To verify the potential clinical relevance of p53-nuclear pore and p53-nuclear lamina axes in PDAC, we examined the expression of the members of these two families in a dataset of nearly 200 PDAC-patients. Interestingly we found that the overall protein expression of the indicated factors was increased in tumour tissue as compared to the normal adjacent counterpart (NAT) (Fig. 5A). Furthermore, selected nuclear lamina and nuclear pore members show increased expression both at mRNA level and protein level in PDAC tissue comparted to the normal counterpart (Fig. 5B, C). Notably, RNA expression of Lmnb1, Nup107, Nup85, Nup205, Nup54 and Nup155 positively correlated with tumour weight, suggesting that their upregulation promotes tumour cell growth or progression (Figs. 5D and 6A). More importantly, we found that Lmnb1, Lmnb2, Nup54 and Nup107 expression is able to consistently stratify patients for prognosis (Figs. 5E, F and 6B). Analysing this axis across several tumour types, we observed a negative correlation between p53 status and the expression of nuclear envelope proteins also in hepatocellular carcinoma (HCC) (Fig. 6C, D), thereby providing evidence for the dysregulation of p53-nucleus crosstalk possibly in other cancer types. Overall, these data provide correlative evidence between p53-nuclear pore and p53-nuclear lamina axes and downstream deregulation of gene expression and rearrangement.

## Conclusions

Our work expands our knowledge about p53-dependent regulatory network highlighting an alternative possible mechanism for p53 mediated gene regulation and control of genomic integrity. Alteration of nuclear metabolism by dysregulation or mutation in genes coding for nuclear lamina and nuclear pore factors have been linked to genetic human disorders such as laminopathies characterized by premature senescence, improper DNA repair process, aberrant transcription and instability of heterochromatinic regions and loss of cell identity [34]. These hallmarks strongly overlap with the transformation process, and the loss of p53 recapitulate in part the effect of the mutation in lamin-coding genes on nuclear metabolism. Furthermore, p53 depletion or mutation might predispose the cell to undergo genomic instability and hence contributing to the cancer evolution [22, 36, 42, 51]. Future work will be required for a comprehensive dissection of p53-nucleus interplay to better define a druggable approach model in the context of human disease and cancer pathogenesis [1], Dal [11]. This will be extremely helpful for the identification of novel potential therapeutic targets and prognostic factors.

## Material and methods

### Cell lines

KPsh cells (kindly donated by Scott W Lowe) derive from PDAC that developed in a *Pdx1-cre;LSL-Kras*^*G12D*^*;Col1a1-TRE-shp53-shRenilla;Rosa26-CAGs-LSL-rtTA-IRES-mKate2* mice [25]. Cells were grown in DMEM (10% FBS Gibco; penicillin–streptomycin 2 units/ml) at 37 °C, 5% CO_2_ and they were propagated on collagen-coated plates (PurCol, Advanced Biomatrix, 0.1 mg/ml). KPsh were maintained in 1 μg/ml doxycycline to keep off p53 through doxycycline-dependent shRNA against *Trp53*; to allow p53 expression the doxycycline was removed 48 h before further procedure. KPsh cells have been authenticated as described in the source papers (references above); we also confirm p53 status by RT-qPCR or western blot.

### Immunofluorescence (IF) and data analysis

Cells were seeded on glass slides, and fixed with Paraformaldehyde (PFA) 4% in PBS (phosphate-buffered saline); fixed cells were permeabilized with Triton™ X-100 (Sigma #T8787) 0.05% for 15’ at RT, and blocked for 1 h with 10% goat serum (Gibco #16210-072); Lamin B1 primary antibody (Santa Cruz #sc-30264) was incubated overnight at 4 °C; slides were washed 3X with PBS and incubated 1 h at RT with the appropriate Alexa Fluor secondary antibody (Thermo Fisher) and DAPI (Sigma Aldrich) to counterstain the nuclei; after washing 3X with PBS slides were mounted with ProLong™ Gold Antifade mounting solution (Thermo #P36934) and acquired in Z-stack mode with confocal microscope (NIKON Eclipse Ti) using EZ C.1 acquisition software (Nikon, Tokyo, Japan); all the images were processed using FIJI software (ImageJ). The fluorescent intensity profiles of Lamin B1 was performed with NIS elements analysis software 6.0 (Nikon).

### Western blot

Cells were lysed with RIPA buffer supplemented with Protease inhibitor cocktail (Roche), the samples were denatured at 98 °C, resolved on a SDS–polyacrylamide gel, and blotted on Amersham Hybond P0.45 PVDF membrane (GE Healthcare Life Science); membranes were blocked by Blotting-Grade Blocker (Bio-Rad) 5% in PBS 0.1% Tween-20, incubated with primary antibodies overnight at 4 °C (anti-p53 Santa Cruz #sc-126; anti-Lamin B1 Santa Cruz #sc-30264, anti-Gapdh #G8795) washed 3X with PBS 0.1% Tween-20, and incubated with the appropriate peroxidase-conjugated secondary antibodies (Bio-Rad) at room temperature for 1 h; detection was performed with the ECL chemiluminescence kit (Perkin Elmer). Densitometry was performed on two biological replicates using the UVITEC ALLIANCE imaging system.

### ATAC-seq and RNA-seq

ATAC-seq and RNA-seq data come from [30] and are deposited under the following GEO accession numbers: GSE207878 (ATAC-seq) and GSE207879 (RNA-seq). Briefly, for ATAC-seq the cell pellet (KPsh ± doxy 48 h, 2 biological replicates) was resuspended in 500 µl of ice-cold cryopreservation solution (50% FBS, 40% growth media, 10% DMSO); for RNA-seq (KPCsh ± doxy 48 h, 3 biological replicates) the RNA was extracted using RNeasy Mini Kit (QIAGEN, #74106) followed by DNaseI step (Sigma, #AMPD1-1KT); Active Motif performed sequencing and bioinformatic analysis. Further details are available at the source paper.

### Bioinformatic analysis

Publicly available datasets from cBioPortal were used to compute correlations analyses of expression (transcriptomics and proteomics) and mutational status (amplification, deletion, mutation) in PDAC (CPTAC, Cell 2021; TCGA, Nature 2020) PanCancer *(ICGC/TCGA, Nature 2020), and HCC (TCGA)* studies. The overall- and disease free- survival analysis were done on GEPIA 2 by searching for the genes of interest in pancreatic cancer and liver hepatocellular carcinoma datasets.

Analysis of the p53 target genes (Fig. 1C–E) was performed using the TargetGeneRegulation database [16].

ChIP-seq data were downloaded from ChIP-Atlas [26, 52] by searching for the DREAM complex member E2f4 binding profile in murine cell lines (*mus musculus*, mm10). The ChIP-seq tracks were extracted and visualized using IGV software.

Lamin B1 and NPC ChIP-seq data are publicly available at GEO datasets GSE96033 [31] and GSE146591 [2], respectively. In both cases the ChIP-seq peaks coordinates were converted in genes list using GREAT [24, 44] online software and the obtained lists were used for further analysis. The DAVID bioinformatic database [18, 41] was interrogated to obtain the chromosome enrichment analysis (Fig. 2F–I) with the list extracted from GREAT.

Gene Ontology (GO) terms enrichment analysis (Fig. 3C and Additional file 1: Fig. S2A, B) was performed by uploading the gene list of interest in CytoScape software [32].

Analysis of genomic loci occupancy (Fig. 3A) was done by analysing the indicated genomic loci using GREAT online software to find their distribution respect the transcription start site (TSS).

### Statistics

The data were analysed by using GraphPad Prism version 8.0.1; the *p*-values were calculated by 2-tailed unpaired and non-parametric t test, unless otherwise mentioned; a *p*-value lower than 0.05 was selected to state statistically significant differences. The z-score values were calculated by applying the following formula: *Z-score* = (*x − µ)/σ*, where *x* = observed sample value, *µ* = mean of the samples, *σ* = standard deviation of the samples.

## Supplementary Information

**Additional file 1: Figure S1** Regulation of nuclear components by p53. **A**–**C** RNA sequencing data analysis of SMC complex (**A**) nucleolus (**B**) and paraspekles (**C**) members. The heatmap (left) and the box plot (right) are shown as z-score and fold change (KP shp53/KP CTR) values, respectively; the orange dotted line is the threshold indicating the genes upregulated or downregulated upon silencing of p53 (shp53). **D** Comprehensive view of datasets for p53-dependent regulation of nuclear pore (left) and the nuclear lamina (right) members. CDKN1A and PLK1 are positive controls for activation and repression by p53, respectively. source: TargetGeneRegulation database [16]. **Figure S2** p53-nuclear envelope dependent genes are frequently mutated in cancer. **A** Pan-cancer oncoprint showing the mutational status of the 15 genes shown in Fig. 4C. Source: TCGA, cBioportal [8]

## Data Availability

Available upon requests.
