# Supplementary figures and images for: p53 regulates expression of nuclear envelope components in cancer cells
Source: Biol Direct. 2022 Dec 2;17:38. doi: 10.1186/s13062-022-00349-3 (PMC9716746; doi:10.1186/s13062-022-00349-3)

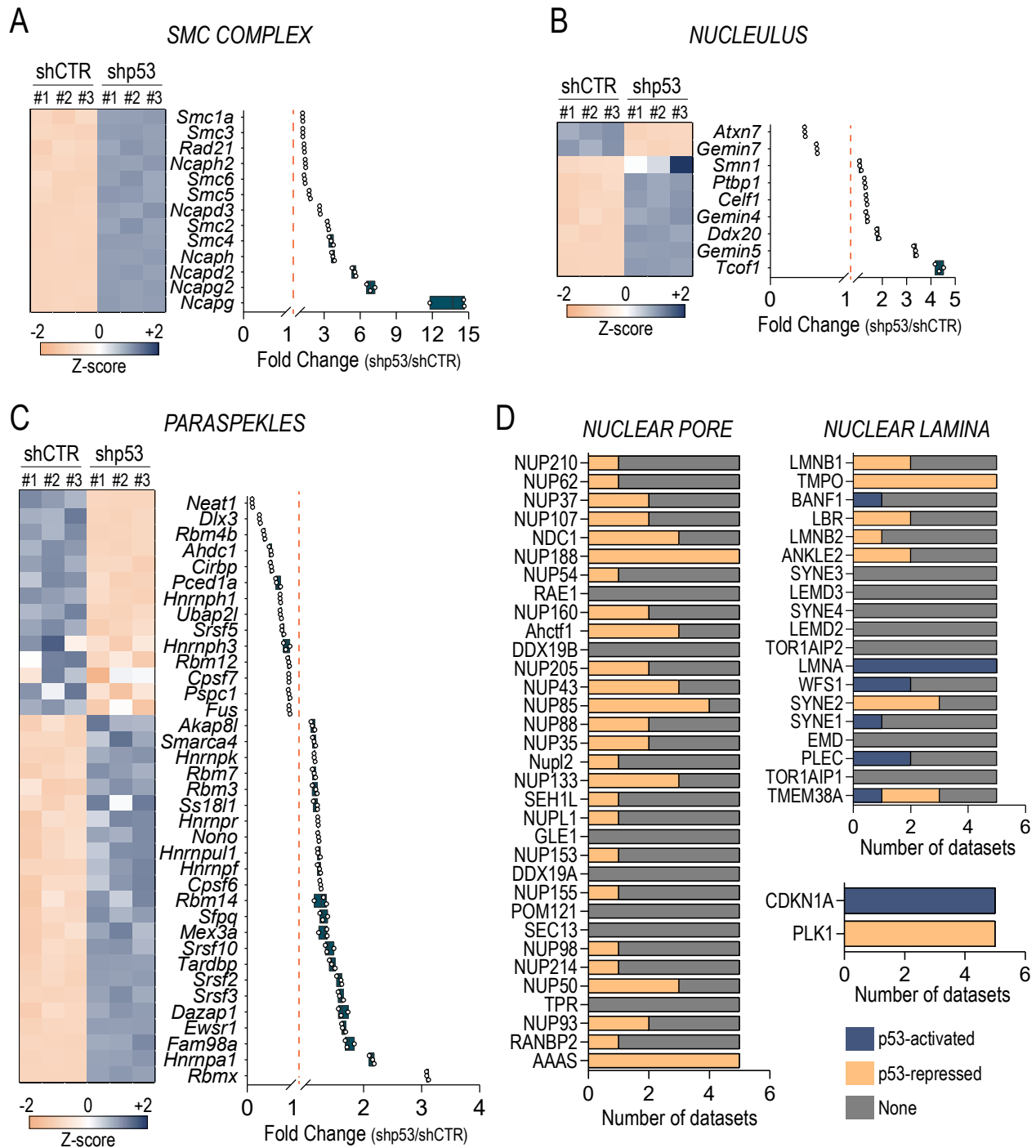

**Supp. Figure 1**

A

*Pan-cancer analysis of whole genomes, 2565 patients (ICGC/TCGA, Nature 2020)*

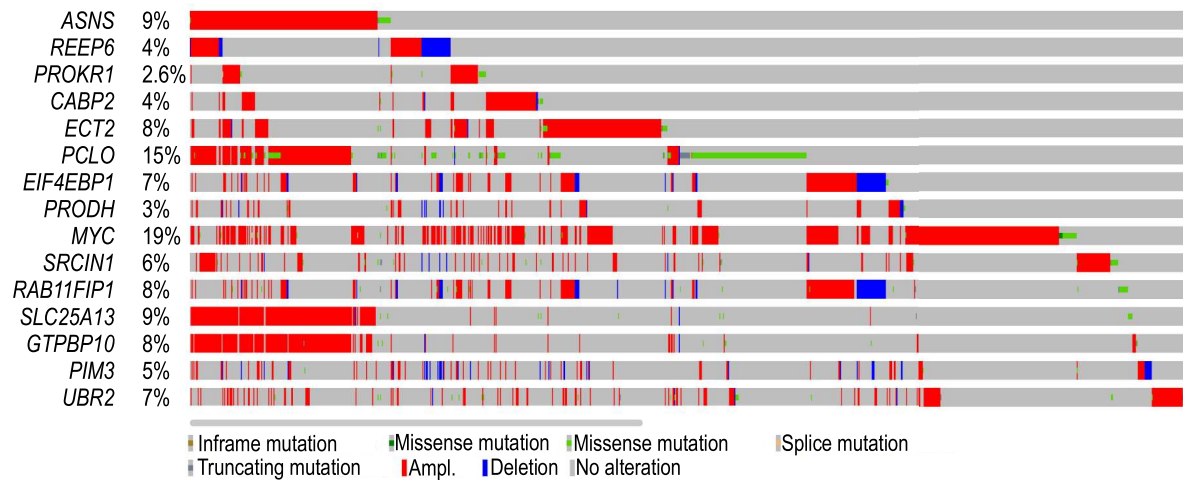

Supplement: Supplementary file 1 — Additional file 1: Figure S1 Regulation of nuclear components by p53. A–C RNA sequencing data analysis of SMC complex (A) nucleolus (B) and paraspekles (C) members. The heatmap (left) and the box plot (right) are shown as z-score and fold change (KP shp53/KP CTR) values, respectively; the orange dotted line is the threshold indicating the genes upregulated or downregulated upon silencing of p53 (shp53). D Comprehensive view of datasets for p53-dependent regulation of nuclear pore (left) and the nuclear lamina (right) members. CDKN1A and PLK1 are positive controls for activation and repression by p53, respectively. source: TargetGeneRegulation database [16]. Figure S2 p53-nuclear envelope dependent genes are frequently mutated in cancer. A Pan-cancer oncoprint showing the mutational status of the 15 genes shown in Fig. 4C. Source: TCGA, cBioportal [8] [file 13062_2022_349_MOESM1_ESM.pdf]
